# Motivated Proteins: A web application for studying small three-dimensional protein motifs

**DOI:** 10.1186/1471-2105-10-60

**Published:** 2009-02-11

**Authors:** David P Leader, E James Milner-White

**Affiliations:** 1 Institute of Biomedical and Life Sciences, University of Glasgow, Glasgow, G12 8QQ, UK

## Abstract

**Background:**

Small loop-shaped motifs are common constituents of the three-dimensional structure of proteins. Typically they comprise between three and seven amino acid residues, and are defined by a combination of dihedral angles and hydrogen bonding partners. The most abundant of these are αβ-motifs, asx-motifs, asx-turns, β-bulges, β-bulge loops, β-turns, nests, niches, Schellmann loops, ST-motifs, ST-staples and ST-turns.

We have constructed a database of such motifs from a range of high-quality protein structures and built a web application as a visual interface to this.

**Description:**

The web application, Motivated Proteins, provides access to these 12 motifs (with 48 sub-categories) in a database of over 400 representative proteins. Queries can be made for specific categories or sub-categories of motif, motifs in the vicinity of ligands, motifs which include part of an enzyme active site, overlapping motifs, or motifs which include a particular amino acid sequence. Individual proteins can be specified, or, where appropriate, motifs for all proteins listed. The results of queries are presented in textual form as an (X)HTML table, and may be saved as parsable plain text or XML. Motifs can be viewed and manipulated either individually or in the context of the protein in the Jmol applet structural viewer. Cartoons of the motifs imposed on a linear representation of protein secondary structure are also provided. Summary information for the motifs is available, as are histograms of amino acid distribution, and graphs of dihedral angles at individual positions in the motifs.

**Conclusion:**

Motivated Proteins is a publicly and freely accessible web application that enables protein scientists to study small three-dimensional motifs without requiring knowledge of either Structured Query Language or the underlying database schema.

## Background

Understanding of the diverse three-dimensional structures of proteins is aided by the recognition of their structural components. The most well-known of these are secondary structure elements, such as α-helix and β-sheet, and the super-secondary structures that can arise from them. Other, smaller, components are also abundant. The first such example was the β-turn [1], which exhibits geometrical constraints at certain of its four residues, and, like secondary structure, is stabilized by hydrogen bonding between peptide bond atoms – in this case a single hydrogen bond. β-turns are structural components in their own right, as they can be defined in terms of their dihedral angles and hydrogen-bond, in the absence of any knowledge of the secondary structure. Recognition of other such abundant small hydrogen-bonded three-dimensional motifs in proteins followed, including the β-bulge [2], the β-bulge loop [3], the αβ-motif [4] and the Schellmann loop [5,6]. These motifs vary in length from three to seven residues, include one or more hydrogen bonds, and are generally associated with secondary-structure features.

Analogous structures to the β-turn occur (so-called side-chain/main-chain mimics) in which the hydrogen bond is between the main-chain NH atom and the side-chain oxygen atom of aspartate or asparagine (asx-turns) or serine or threonine (ST-turns) [7,8]. Other frequently occurring motifs involving side-chain hydrogen bonds were identified: asx-motifs [9], ST-motifs [10] and ST-staples [11]. There are also some abundant small motifs which involve the interaction of pairs of main-chain NH or CO groups – often by hydrogen bonding – with cationic or anionic groups, respectively. Examples of these are the nest [12] and the niche [13].

We have constructed a relational database of these motifs that can be interrogated using Structured Query Language (SQL). To allow protein scientists who may be unfamiliar with SQL to access this database. we have built an associated web application, entitled 'Motivated Proteins'. This web application allows results to be visualized in a variety of ways, most importantly in the context of the three-dimensional structure of the protein. It is designed to facilitate specific queries from protein scientists whose focus is a particular protein or motif, but also lets protein scientists without such a focus explore this area of protein structure.

## Construction and content

### Choice and definition of motifs

The database currently includes the twelve motifs mentioned in the Background section, above, using the criterion for inclusion that at least 2% of the amino acid residues in proteins belong to a particular motif. These twelve categories are divided into a total of 48 sub-categories (Additional file 1) on the basis of certain features. These features include specific variations in length (e.g. Schellman loops can be seven or eight residues in length), defining amino acid side-chain (e.g. S or T for S/T turns) and, in the specific case of β-bulges, whether the non-contiguous hydrogen-bonding partner is on the *N*-terminal side of the pair. In addition, different defining dihedral angles in the sub-categories may arise in two ways. The first is where there are alternative forms of a motif produced by peptide-plane flipping [14]. The second is where there is an alternative enantiomeric form of the backbone of certain of the residues. (These give rise to the 'Flipped' and 'Reflected' attributes, respectively, in Additional file 1).

Other facilities that provide information on small protein motifs use the categories described here, although they do not currently employ this sub-categorization in full [15,16].

### Database design, implementation, and population

**Figure 1:**
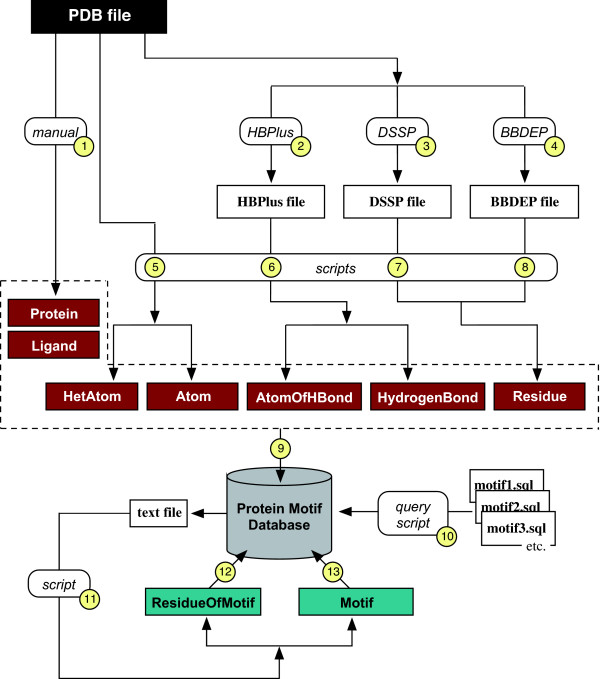
**Schematic diagram of pipeline for constructing the database**. The order of operations is indicated numerically. Database tables are shown with coloured backgrounds: primary entities (those with attributes derived directly by processing information in PDB files) are in claret (darker), entities derived by querying the primary entities and their relationships are in blue-green (brighter). Intermediate text files are not generally shown, nor is the manual addition of active site data to the residue entity, or the pruning of some of the type-1 β-turns described in the text.

Because of the disparate size of motifs and the diversity of their defining features, we have adopted a database schema in which these features are not incorporated into a motif entity itself. Rather, they are embodied in the relationship of such a motif entity to an amino acid residue entity, and in the relationship of this residue entity to entities representing the atoms and hydrogen bonds of a protein. Thus, the database is fundamentally one that models the protein – the motifs are derived from this 'core database' by SQL queries, and then added to it. Full details of the database schema and tables are available as Additional files 2 and 3 – here we describe the construction pipeline for the key information in the database (Fig. 1):

The starting point for generating the tables of protein data was a set of 500 PDB (Protein Data Bank) files prepared by the Richardson laboratory [17]. The reason for using these files was that the coordinates are of high quality, they include hydrogen atoms and corrected side-chain amide atom positions, and have been edited so that in oligomers only one subunit is represented. Some further editing of these files was necessary: where alternative conformations are listed for individual residues, only the first was retained. Of the 500 proteins, 417 were included in the database, supplemented by twelve from the PDB chosen to broaden the coverage of protein folds (Additional file 4).

In Fig. 1 it can be seen that the edited PDB files were the source of the coordinate data in the 'Atom' and 'HetAtom' entities. Processing the PDB files with the program HBPlus [18] generated the hydrogen-bond data for the 'HydrogenBond' entity. Processing the PDB files with the program DSSP [19] generated the φ and ψ dihedral angles and the secondary structure designations of the Residue entity. Processing the PDB files with the program BBDEP [20] generated the χ^1 ^and χ^2 ^angles of the Residue entity.

Perl scripts were written to automate processing, but generation of some tables required manual intervention. Population of the table for the 'Ligand' entity (Fig. 1) required subjective assessment of the functional relevance of the entries in the 'HetAtom' table (corresponding to 'HETATM' lines in the PDB file). The data for the table describing the 'Protein' entity were prepared by hand to allow inclusion of EC (Enzyme Commission) numbers, and to allow consistency in nomenclature for indexing. Active site data for the table describing the 'Residue' entity were obtained by consulting the Catalytic Site Atlas at EBI, Cambridge [21], and added manually.

SQL queries were written to harvest each sub-category of motif from this initial database, and Perl scripts written to automate the generation of the table describing the 'Motif' entity and the 'ResidueOfMotif' relationship entity from these queries (Fig. 1). Some of the type I β-turns obtained in this manner qualify as such only because they are parts of α-helices or 3_10 _helices. These were removed from the database after running SQL queries to identify them. The 48 motif-defining SQL queries are provided as Additional file 5. The definitions of motif sub-categories are given in a Motif Glossary within the web application. Although a specific query is provided in the Motivated Proteins web application for searching for instances of any these 48 sub-categories, simplified motif menus of the twelve main categories are employed in other queries (*see *Utility section, below).

The database is implemented in the MySQL relational database management system (version 5.0.41) and has been deployed on servers variously running the Solaris, Linux or Mac OS X operating systems. We refer to the database as the 'Protein Motif Database', a name which distinguishes it from applications we have written that provide access to it, including the Motivated Protein web application which is described below.

### Construction of the Motivated Proteins web application

We have used Java servlet technology to provide web access to the Protein Motif database, the servlet currently running in a Sun Java System Web Server (version 7.0) on the same machine as the Protein Motif database. It generates the main XHTML query pages (level 1.0 Strict) with which the user interacts (Utility section).

In servlet-based web applications, new pages are generated in a linear manner as a result of essentially form-based queries. In Motivated Proteins some such queries populate menus on resulting pages from which the user makes choices to formulate a scientific query which, in turn, returns a page containing the results. This latter is furnished with a form from which further queries can be made. Where alternative views of data – or supplementary information – are invoked by the user, they have been taken out of this linear query stream as small 'pop-up' web pages, generated by CGI applications written in Perl.

Three-dimensional structural visualizations in the 'pop-up' pages employ the Jmol Java applet [22] and associated JavaScript library, supplemented with some custom JavaScript functions to co-ordinate the behaviour of the controls. Bitmap graphics visualizations were generated dynamically from query data embedded as CGI parameters in links on the pages. Some (the histograms and secondary structure representations) were created by CGI applications using Lincoln Stein's Perl GD module [23]. In one case in which there were too many data to include in a URI to a CGI application (the dihedral angle plots) a 'headless' Java servlet was used.

Population of alphabetical protein indexes was done dynamically using AJAX and a separate Java servlet.

## Utility

**Figure 2:**
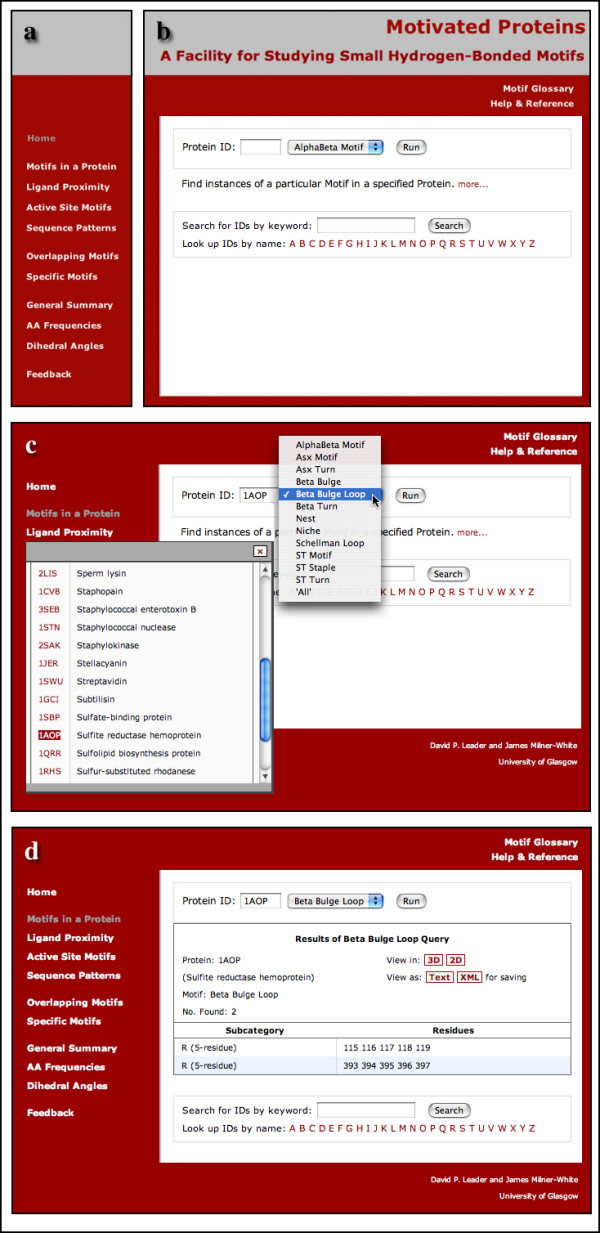
**Construction of queries in the Motivated Proteins web application**. (a) The navigation menu; (b) Typical query page; (c) Selection of motif and PDB identifier; (d) Typical results page. Version 3 of the Safari web browser was used.

The Motivated Proteins web application presents the user with a menu of options, at the left-hand side of each page. At the top is 'Home' and at the bottom 'Feedback', leaving the database queries in three groups in the middle. Of these, four queries can be regarded as primarily 'protein-based', two can be regarded as more 'motif-based', and three are summary queries (Fig. 2a).

### 'Protein-based' Queries

Three of this first group of queries involve specifying a protein and selecting a specific category of motif (Fig. 2b and 2c). Depending on the query type, one obtains all instances of the chosen motif in the protein, those within 4Å of a ligand, or those which include enzyme active-site residues. The fourth query allows searches for the occurrence of a short amino acid sequence string within a specific motif or all motifs. In the latter case the number of 'hits' displayed can be restricted.

### 'Motif-based' queries

The first query in the second group allows searches for overlaps between two types of motif. In this case there are two drop-down menus of motifs, the second with an 'All' option, which the user is advised to employ in an initial screening step because the large number of possible combinations will generally include many that are not represented in any individual protein. The second query allows the user to retrieve instances of any of the 48 sub-categories of motif. This is useful for systematic work, especially when one wants to locate examples of less abundant motifs. (One can make a preliminary summary query – below – to determine the abundance of the different sub-categories.)

### Presentation of the results of non-summary queries

The initial results of the queries described above are presented as tables in a page which the user can print or save (Fig. 2d). The XHTML-compliance of the pages allows them to be parsed as XML. However, as the XHTML tends to be rather extensive, links to other machine-readable textual options are provided (Fig. 2d). One of these is an easily-parsable plain-text format and the other is custom XML for which a DTD (Document Type Definition) has been created.

**Figure 3:**
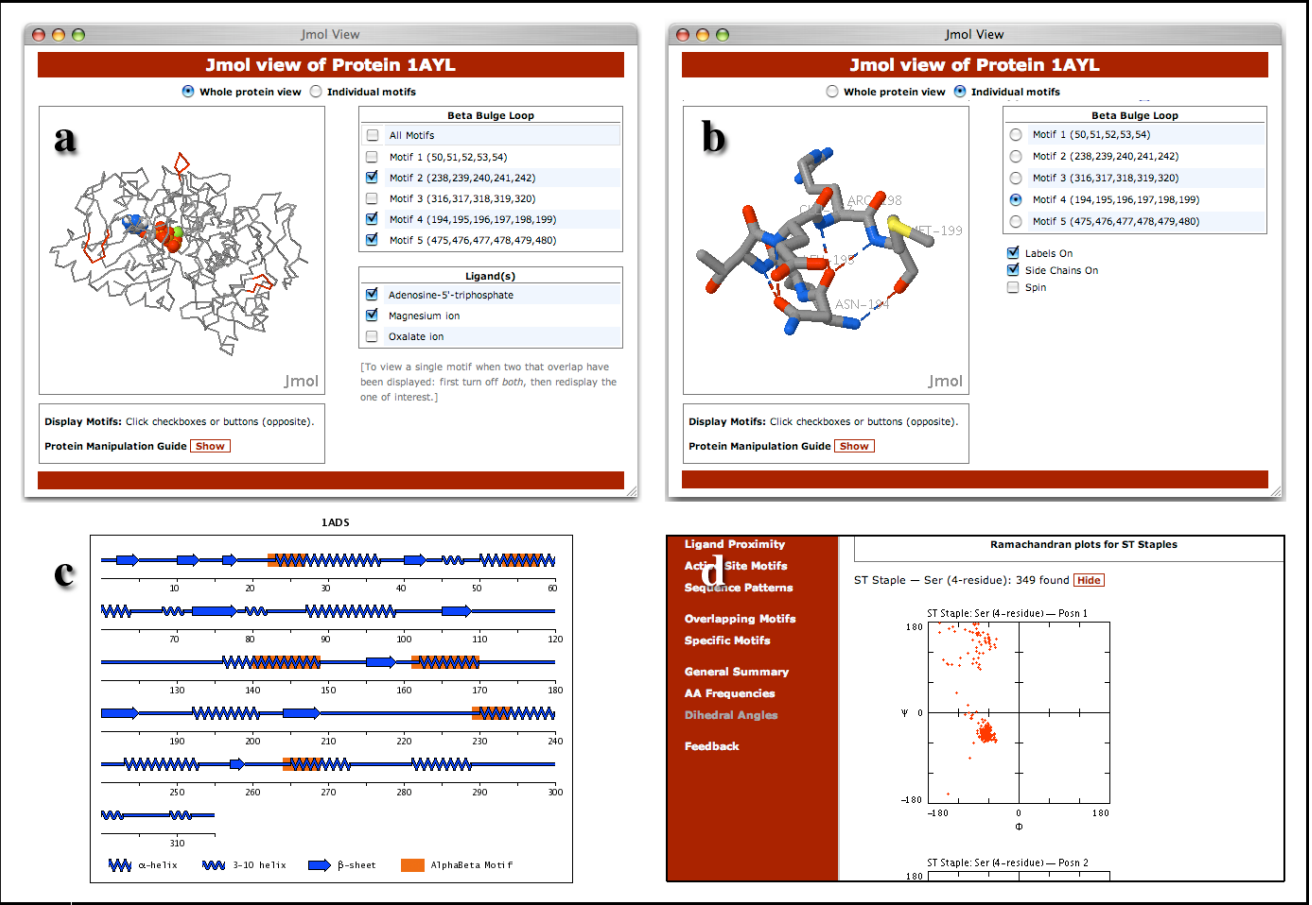
**Additional data views**. (a) Default view of β-bulge loops in the three-dimensional structure of phosphoenolpyruvate carboxykinase, after user selection of features; (b) Three-dimensional view of an individual β-bulge loop from (a); (c) Secondary structure cartoon of αβ-motifs in aldose reductase; (d) Section of a page displaying summary results for dihedral angles at different positions in ST Staples. Data for the first position in one of the sub-categories of this motif can be seen.

A key feature of Motivated Proteins is the use of the open-source, cross-platform Jmol viewer to visualize motifs in the context of the three-dimensional structure of the protein. For queries restricted to one protein, a link labelled '3D' (Fig. 2d) invokes a window containing a protein model in which the motifs can be visualized. For queries that return a list of motifs from different proteins, each item in the list has its own link to invoke a view of that motif alone in the context of the protein tertiary structure. Fig 3a shows an example of such a view. One has the option of using buttons to display the motifs in colour, and, where relevant, any of the associated ligands. One can also switch to a view of individual motifs, which are presented with the side-chains and hydrogen-bonds displayed (Fig. 3b). It should be emphasized that all hydrogen bonds involving residues in the motifs are presented – whether or not they define the motif – and that these are loaded from the database (i.e. they are originally derived from running the HBPlus program on the protein). This provides a useful perspective on the environment of motifs.

For the queries which find individual or overlapping motifs in a protein, there is the option (labelled '2D' – Fig. 2d) of viewing the motifs in the context of a graphic of the primary structure of the protein showing a simple cartoon representation of the secondary structure (Fig. 3c). An original Perl script (SecondGlance) is used to generate these graphics, which are based on those of the Wirplot diagram [24].

### Summary queries

Three different types of summary query are provided. The 'General Summary' section provides access to tables listing the number of each sub-category of motif, and the number of each category of motif in the proximity of a ligand or including an active site residue. These tables are populated dynamically from the database. For overlapping motifs, the main overlapping partners for each motif are listed in a text page. The 'AA Frequencies' section provides histograms for the occurrence of the amino acids at each position of a motif sub-category. The 'Dihedral Angles' section allows the user to generate and view φ/ψ plots for each position in each sub-category of a selected motif (Fig. 3d).

## Discussion

A point of particular concern in designing Motivated Proteins was to avoid placing the user in situations which might dispose him to abandon the web application unnecessarily. This will be discussed in the context of two general ways in which we envisage the resource being used.

The first way in which we envisage the resource being used is by a protein scientist with a focus on a particular protein or motif. A potential problem here is that a protein of interest might not be present in the database, given that it was constrained in size for the reasons described in the Construction and Content section, above. The application is designed so that under such circumstances an external call is made to the CATH facility at University College, London [25] to retrieve the CATH structural classification code of the query protein. A search is then made of the local database for the proteins with CATH codes closest to this, and these are presented as options to the user. As the Protein Motif Database provides coverage of the first two levels (Class and Architecture) of the CATH classification there is a good chance that a structurally related protein will be found. In the event that the query protein has not received a CATH classification, an external call is made to the PDBSum SearchHeaders.pl facility at EBI, Cambridge, and functionally related alternatives are offered.

The second way in which we envisage the resource being used is by a protein scientist who wishes to explore these structural motifs, without having a particular protein or motif in mind. A potential problem here is the need to specify the PDB identifier of a protein example. For this reason an alphabetic index of the names of proteins in the database is provided (Fig. 2b). Selecting a letter of the alphabet invokes a floating list of corresponding protein names and PDB identifiers, and clicking on one of the latter enters it in the search field (Fig. 2c). (This index is context-sensitive – if one is searching for motifs near a ligand, for example, only those proteins with a ligand are included.) Alternatively, a keyword search can be performed to find proteins in the database answering a specific description (Fig. 2b–d).

In both cases considered above it can happen that the user makes a query for a motif, only to find that there are no instances of that motif in the protein selected. For this reason the pull-down menu for selecting motifs has an option, 'All' (Fig. 2c), which, on running, returns a listing of the number of motifs of each category in the specified protein. This listing can then form the basis for fruitful queries on the protein. If the focus is a particular motif, the user is able to employ the 'Specific Motifs' menu option.

## Conclusion

We believe that the public availability of Motivated Proteins will assist scientific research on small hydrogen-bonded three-dimensional motifs within proteins, and hope that it may also lead to a greater appreciation of the occurrence and potential importance of such motifs.

## Availability and requirements

### Availability

The URI of the Motivated Proteins site is , with direct access to the web application at . The web application is publicly and freely accessible, requiring no registration and with no restrictions on use. All server scripts and Java source code supporting the web application are available, on request, under the GNU General Public License.

### Requirements

The basic features of Motivated Proteins only require that the web browser support JavaScript and CSS, but display of three-dimensional structures using the Jmol applet imposes a requirement for support of Java 1.4 and LiveConnect. Qualifying web browsers include Internet Explorer 6 and 7 and Chrome on Windows, Firefox (1.0 and above) and Opera (7.5.4 and above) on a variety of platforms, and Safari (1.2 and above) on Mac OS X (10.3.3 or greater) or Windows.

## Authors' contributions

The need for a database arose from EJMW's work on many of the motifs mentioned here. The idea of a web application supported by a relational database emerged from both authors, with DPL responsible for software design and implementation and made the initial design of the web interface. Both authors contributed to the writing of the manuscript, with DPL making the initial draft. Both authors read and approved the final manuscript.

## Supplementary Material

Additional file 1 **Motif sub-category definitions.** The file contains the output of an SQL query to list the whole of the MotifDescription Table (Additional file 2). It should be viewed in a mono-spaced font such as Courier. The description of the attributes for this entity are included in Additional file 3. Click here for file

Additional file 2 **Schema of the Protein Motif Database underlying the Motivated Proteins web application.** This file shows a standard entity-relationship diagram of the main entities in the database, excluding views and entities related to CATH classification and Keywords. Primary entities (those with attributes derived directly by processing information in PDB files) are in claret (darker). Entities derived by querying the primary entities and their relationships are in green (brighter). Click here for file

Additional file 3 **Entities in the Protein Motif Database.** This file contains the output of SQL queries to describe the database and the entities in tabular form. It should be viewed in a mono-spaced font such as Courier. The listing also includes tables for views and minor entities not included in the diagram in Additional file 2. Click here for file

Additional file 4 **Proteins in the Protein Motif Database.** This file lists the PDB identifiers of the 429 proteins in the Protein Motif Database. The twelve entries not derived from the Richardson 'Top 500' are indicated. Click here for file

Additional file 5 **SQL queries to retrieve motifs from protein data in the database. **This is a zipped directory containing 48 plain text files of SQL queries for the different sub-categories of motif used to populate the Protein Motif Database. Click here for file
