# Supplementary figures and images for: Motivated Proteins: A web application for studying small three-dimensional protein motifs
Source: BMC Bioinformatics. 2009 Feb 11;10:60. doi: 10.1186/1471-2105-10-60 (PMC2651126; doi:10.1186/1471-2105-10-60)

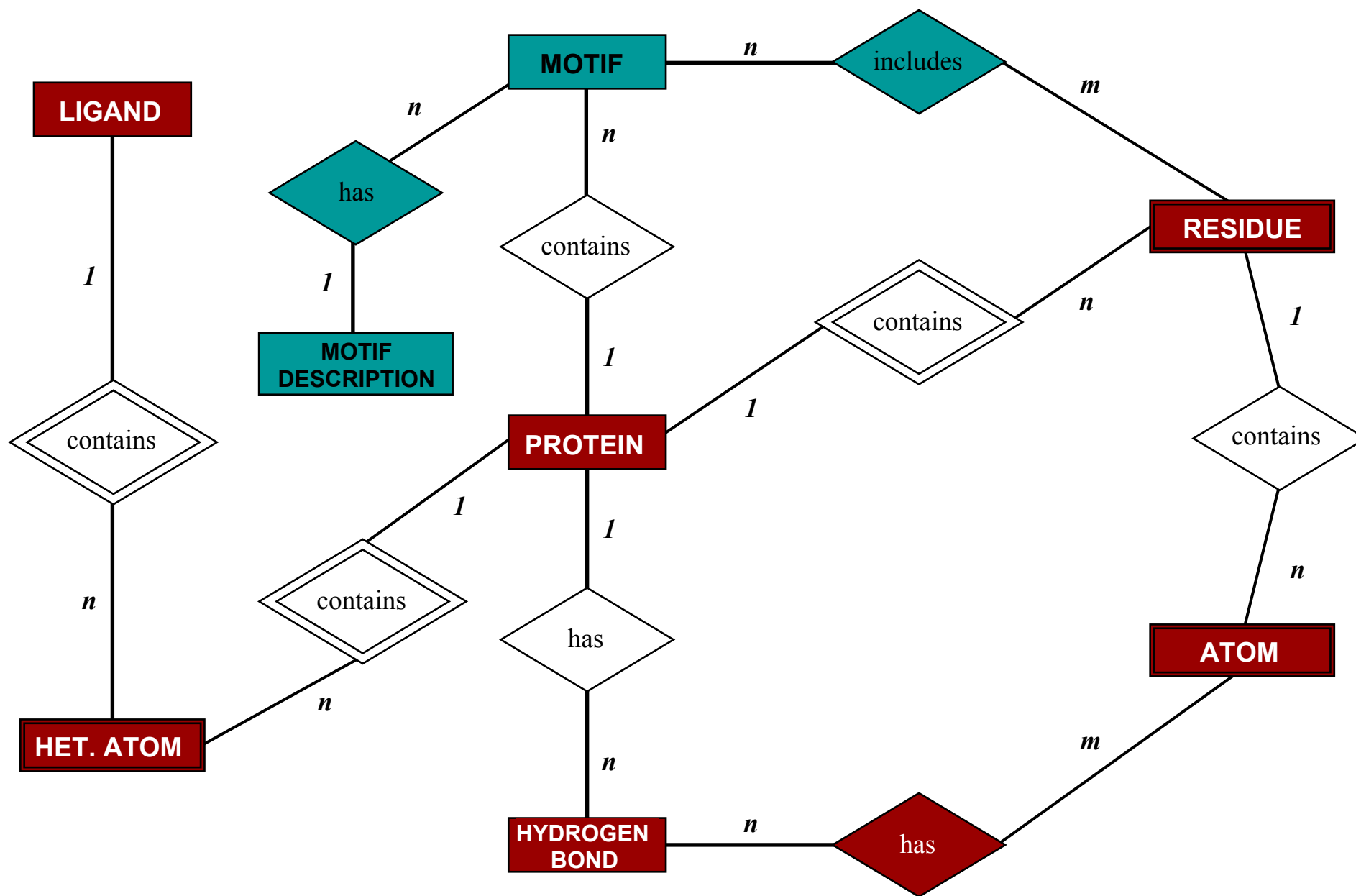

Supplement: Additional file 2 — Schema of the Protein Motif Database underlying the Motivated Proteins web application. This file shows a standard entity-relationship diagram of the main entities in the database, excluding views and entities related to CATH classification and Keywords. Primary entities (those with attributes derived directly by processing information in PDB files) are in claret (darker). Entities derived by querying the primary entities and their relationships are in green (brighter). [file 1471-2105-10-60-S2.pdf]
